# CT-derived body composition analysis could possibly replace DXA and BIA to monitor NET-patients

**DOI:** 10.1038/s41598-022-17611-3

**Published:** 2022-08-04

**Authors:** Lennard Kroll, Annie Mathew, Giulia Baldini, René Hosch, Sven Koitka, Jens Kleesiek, Christoph Rischpler, Johannes Haubold, Dagmar Fuhrer, Felix Nensa, Harald Lahner

**Affiliations:** 1 Institute for Diagnostic and Interventional Radiology and Neuroradiology, University Hospital Essen, Essen, Germany; 2 Department of Endocrinology, Diabetes and Metabolism and Division of Laboratory Research, University Hospital Essen, Essen, Germany; 3 Institute for Artificial Intelligence in Medicine, University Hospital Essen, Essen, Germany; 4 Department of Nuclear Medicine, University Hospital Essen, Essen, Germany

**Keywords:** Endocrine system and metabolic diseases, Diagnostic markers, Whole body imaging

## Abstract

Patients with neuroendocrine tumors of gastro-entero-pancreatic origin (GEP-NET) experience changes in fat and muscle composition. Dual-energy X-ray absorptiometry (DXA) and bioelectrical impedance analysis (BIA) are currently used to analyze body composition. Changes thereof could indicate cancer progression or response to treatment. This study examines the correlation between CT-based (computed tomography) body composition analysis (BCA) and DXA or BIA measurement. 74 GEP-NET-patients received whole-body [68Ga]-DOTATOC-PET/CT, BIA, and DXA-scans. BCA was performed based on the non-contrast-enhanced, 5 mm, whole-body-CT images. BCA from CT shows a strong correlation between body fat ratio with DXA (r = 0.95, ρC = 0.83) and BIA (r = 0.92, ρC = 0.76) and between skeletal muscle ratio with BIA: r = 0.81, ρC = 0.49. The deep learning-network achieves highly accurate results (mean Sørensen-Dice-score 0.93). Using BCA on routine Positron emission tomography/CT-scans to monitor patients’ body composition in the diagnostic workflow can reduce additional exams whilst substantially amplifying measurement in slower progressing cancers such as GEP-NET.

## Introduction

Monitoring the body composition of patients with slow proliferating cancers, such as well-differentiated neuroendocrine tumors (NET), is crucial for predicting the course of the disease and adapting therapy on an individual level^1,2^. Several methods for assessing body composition (BC) are currently used to assess nutritional status, such as dual-energy X-ray absorptiometry (DXA), bioelectrical impedance analysis (BIA) and computed tomography (CT). BC measurements are used to visualize the distribution of different tissue types in the patient. Changes in an individual's BC, whether abrupt or slow, can indicate tumor growth, metastatic spread or therapy response and tumor regression. It is therefore important to monitor BC throughout the disease process to detect these changes and treat the patient accordingly.

DXA has become the method of choice to monitor BC in patients with NET due to its reliable and quickly obtained results. The two low-energy levels used in DXA allow total body adipose tissue, muscle mass, bone mineral content and bone mineral density to be distinguished from each other^3^.

DXA is fast for the subject and the operator. A typical whole-body scan takes approximately 5 to 15 min and exposes the subject to 4–5 µSv of radiation. Mathematical algorithms allow the calculation of body composition using various physical and biological models^4^.

BIA uses electrical current rather than radiation. The analysis of body composition via BIA produces estimates of total body water (TBW), fat-free mass (FFM), skeletal muscle mass (SMM) and fat mass by measuring the body’s resistance as a conductor to an electrical current. Fat mass (FM) is measured by subtracting FFM from total mass^5^.

Computed tomography (CT) is one of the most used imaging techniques worldwide, with more than 70 million scans in the US and over 12 million scans in Germany performed each year (2017)^6,7^ (http://ec.europa.eu/eurostat/statistics-explained/index.php?title=Healthcare_resource_statistics_-_technical_resources_and_medical_technology, last accessed: 01/30/22). While CT scans offer an immense amount of image information, only a fraction is used for diagnostic purposes. Using deep learning networks, the CT data can be used to perform a whole-body composition analysis (BCA) within the routine staging, which would otherwise require significantly more time if the segmentation were executed by a human reader.

Regular sectional imaging via SSTR-PET/CT-scans (somatostatin receptor positron emission tomography/computed tomography) is recommended for diagnosis and during treatment, as well as follow-up-treatment of patients with advanced NET, according to international clinical guidelines^8^. SSTR-PET/CT-scans use radioligands such as [68Ga]-DOTATOC to visualize increased SSTR-expression in the patient’s body as an indication of a NET^9^.

Thus, CT-imaging is a component of PET/CT-scans and is obtained in the routine process. Cutting out additional radiation dose and up to two additional examinations by employing BCA into the diagnostic algorithm would be an improvement to patient care due to its generalizable, time and resource-efficient workflow compared to non-automated BC software.

Consequently, the purpose of this study is to analyze if BCA can achieve comparable results as DXA or BIA-based BC in the clinical treatment of NET patients.

## Results

Seventy-four consecutive, unique patients treated between February 2019 and October 2021 with histologically confirmed, well-differentiated NET were identified in the in-house, prospective NET database. 36 subjects (48.65%) were female, 38 (51.35%) male (Table 1). The median age of subjects with different types of gastro-entero-pancreatic (GEP)-NET at baseline was 63.7 ± 11.5 years. At the time of the data cut-off, 7 patients had died.

**Table 1:** Patient characteristics and results of body composition measurement assessed by BCA, DXA and BIA (n = 74).

(Stated in mean ± standard deviation)	Healthy weight (n = 22)	Overweight (n = 35)	Obesity (n = 17)
Male sex (n)	10	20	8
Female sex (n)	12	15	9
Age (years)	65 ± 10.59	66.11 ± 11.21	60.11 ± 12.87
Weight (kg)	64.92 ± 9.56	79.58 ± 11.28	99.11 ± 9.68
Height (cm)	171.31 ± 9.16	170.57 ± 10.72	173.58 ± 8.48
BMI (kg/m^2^)	22.04 ± 1.95	27.22 ± 1.42	32.96 ± 3.48
BCA: BFR (%)	32.43 ± 11.25	40.36 ± 7.04	49.79 ± 7.65
DXA: BFR (%)	29.27 ± 9.99	35.02 ± 7.38	41.36 ± 6.88
BIA: BFR (%)	28.85 ± 7.61	35.34 ± 7.91	39.83 ± 6.54
BCA: SMR (%)	27.84 ± 4.9	26.57 ± 4.19	23.65 ± 5.17
BIA: SMR (%)	34.11 ± 5.97	32.15 ± 5.05	30.82 ± 3.65

All 74 patients received regular PET/CT-scans for staging, out of which 67 patients received a subsequent DXA-scan. 52 of the total patients received BIA-scans. BIA-scanning was discontinued during the course of the study due to the high correlation between DXA and BIA-based BC (r = 0.92, ρC = 0.76 on overall analysis) in order to spare an additional examination for the partially immobile patients. One scan of each modality was used for the intermodal comparison, amounting to 52 patients who received all three exams and 15 patients who received only PET/CT and a DXA-scan. No patient received only a PET/CT in combination with a BIA-scan.

### Deep learning-network performance

**Figure 1:**
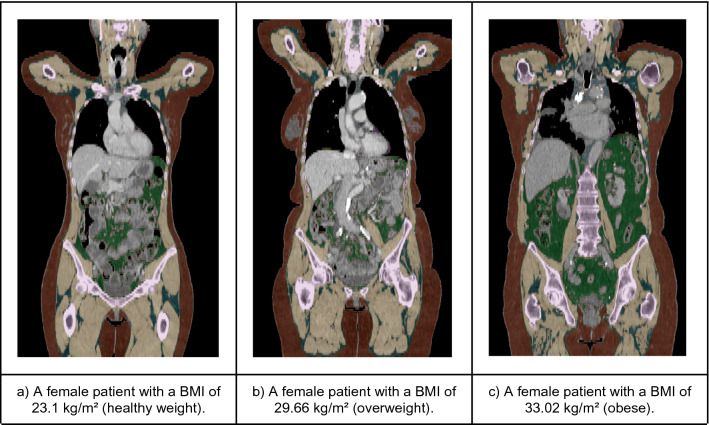
Exemplary full tissue analysis segmentations generated by the BCA network, gathered from patients out of the three BMI-groups. The segmentation shows seven tissues: Muscle (beige), bone (pink), subcutaneous adipose tissue (red), visceral adipose tissue (green), intermuscular adipose tissue (teal), paracardial adipose tissue (light blue), epicardial adipose tissue (purple). The coronal views also illustrate the described patient positioning requested by the CT protocol.

The in-house deep learning system produced highly accurate predictions of all segmented body regions on the independent test-dataset (“Deep learning architecture” section). The Sørensen Dice Score for relevant semantic body regions calculated on the test set amounted to a mean score of 0.93 and the following results on the differentiated regions: thoracic cavity: 0.97, mediastinum: 0.86, pericardium: 0.96, subcutaneous tissue: 0.96, muscle: 0.94 and abdominal cavity: 0.97. Subsequently, a fully automated BCA was performed on the complete study cohort using the trained system (Fig. 1). The mean processing time amounted to 100.63 s per scan using the NVIDIA TITAN RTX.

### Biomarker-extraction

As shown in Table 1, the two main parameters for cancer patient-related BC measurements, Skeletal muscle ratio (SMR) and body fat ration (BFR), were extracted using the three different methods (e.g., “Automated tissue-quantification” section). The correlation analysis showed a strong correlation between BCA and DXA with respect to the BFR (r = 0.95, ρC = 0.83) measured over all BMI-groups (body mass index).

The overall comparison between the BCA and BIA analyses showed equally strong results (r = 0.92, ρC = 0.76), and BIA vs. DXA showed a similar consistency in overall BFR: r = 0.93, ρC = 0.93.

The SMR can be compared between BCA and BIA. With all patients included, a strong correlation between these two methods can be observed: r = 0.81, ρC = 0.49.

Furthermore, the non-correlation test for Pearson’s r showed that the correlation between all groups is significant (p < 0.001). The Shapiro–Wilk test confirmed that the BCA, DXA and BIA measurements grouped by BMI category look Gaussian (p-values between 0.17 and 1.0).

### Body fat ratio

**Figure 2:**
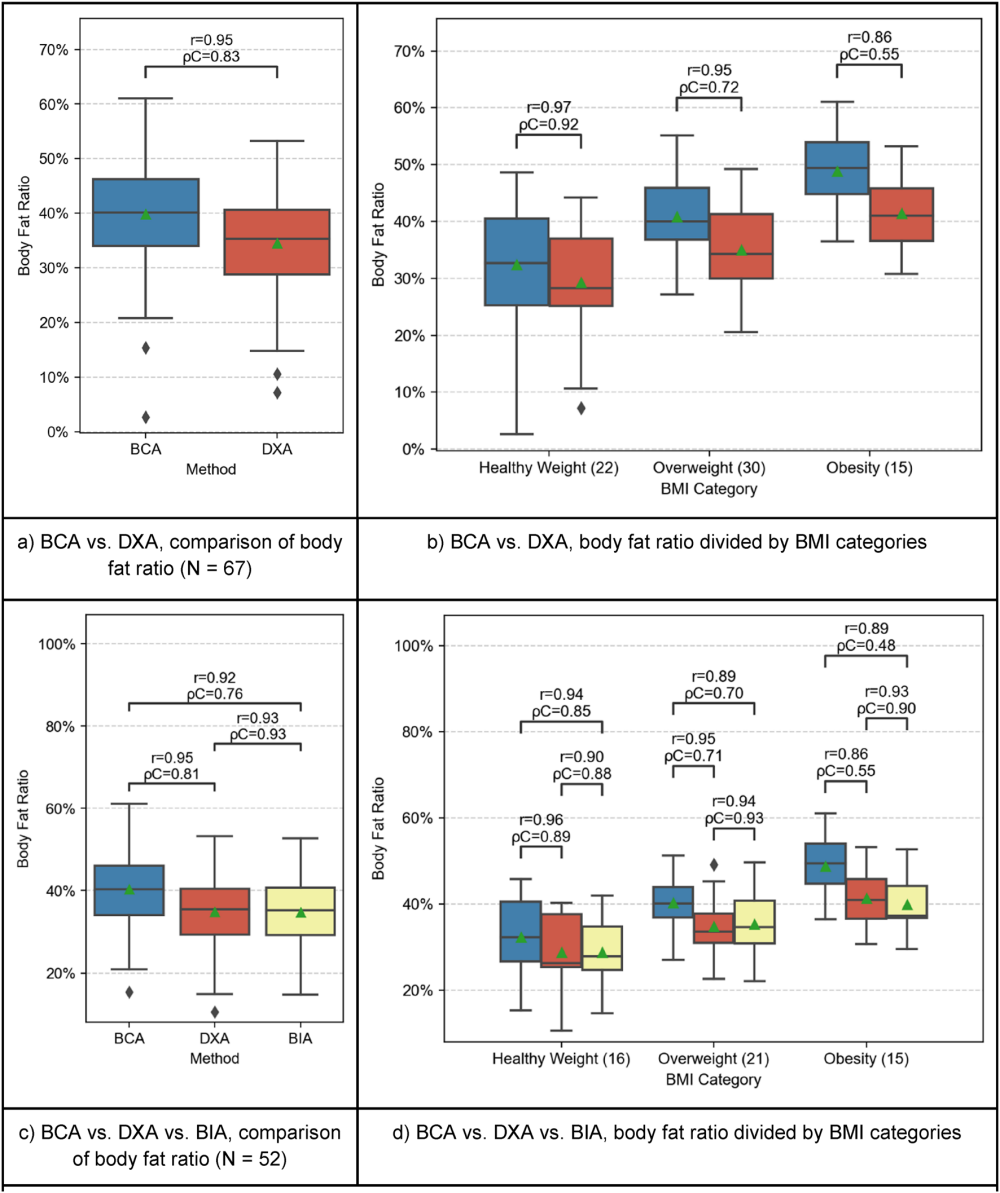
Comparison of BFR between BCA (blue), DXA (red) and BIA (yellow). In the plots (**a**,**b**), BCA and DXA are compared separately because more patients received PET/CT- and DXA scans. Patients with all three measurements available are compared in plots (**c**,**d**). The boxplots represent the distribution of the patient’s BC measurements. The mean is indicated with a green triangle and the outliers are indicated with a rhombus. The samples are compared using Pearson’s r correlation coefficient (r) and Lin’s concordance correlation coefficient (ρC).

As shown in Fig. 2, BCA generally showed a strong correlation with DXA-scans with respect to BFR (r = 0.95, ρC = 0.83). However, there are differences in the subgroups.

Strong results were found for healthy weight patients and overweight patients (BMI-group 1: r = 0.97, ρC = 0.92; BMI-group 2: r = 0.95, ρC = 0.72), but slightly weaker results were found for obese patients (BMI-group 3: r = 0.86, ρC = 0.55).

This trend continues when comparing BCA with BIA (Fig. 2): strong positive correlations were found for all patient groups, though the correlation for overweight and obese patients was slightly weaker than that of healthy patients (BMI-group 1: r = 0.94, ρC = 0.85.; BMI-group 2: r = 0.89, ρC = 0.70; BMI-group 3: r = 0.89, ρC = 0.48).

Figure 4 visualizes these relations as Bland–Altman plots and shows that the mean difference between each method and BCA is approximately 5% (5.29 ± 0.8% for BCA vs. DXA, 5.67 ± 1.12% for BCA vs. BIA). Obese patients tend to differ more in the intermodal comparisons of both DXA and BIA with BCA (Fig. 4a,b), while the plot for the healthy weight-cohort displays a smaller difference to the mean. To visualize potential sex-related differences in BFR, the study cohort was itemized into two sex-related groups. The three methods were compared and show clearly positive correlations between BCA vs. DXA (males: r = 0.95, ρC = 0.78; female: r = 0.95, ρC = 0.81), BCA vs. BIA (males: r = 0.93, ρC = 0.64; females: r = 0.91, ρC = 0.77) and DXA vs. BIA (males: r = 0.92, ρC = 0.91; females: r = 0.91, ρC = 0.91) as shown in Supplementary Fig. 3.

### Skeletal muscle ratio

**Figure 3:**
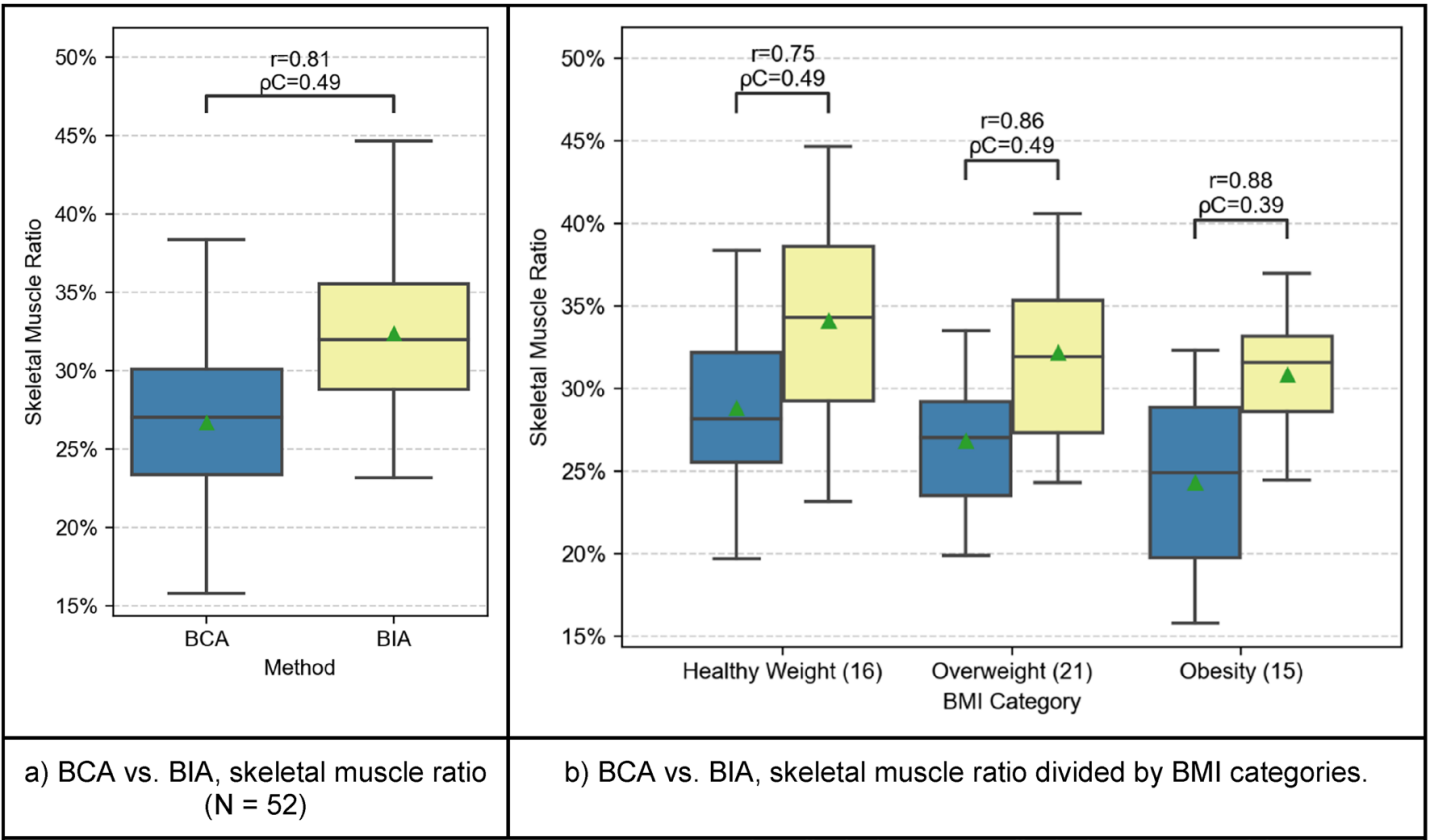
Comparison of SMR between BCA (blue) and BIA (yellow). The boxplots represent the distribution of the patient’s measurements. The mean is indicated with a green triangle and the outliers are indicated with a rhombus. The samples are compared using Pearson’s r correlation coefficient (r) and Lin’s concordance correlation coefficient (ρC).

**Figure 4:**
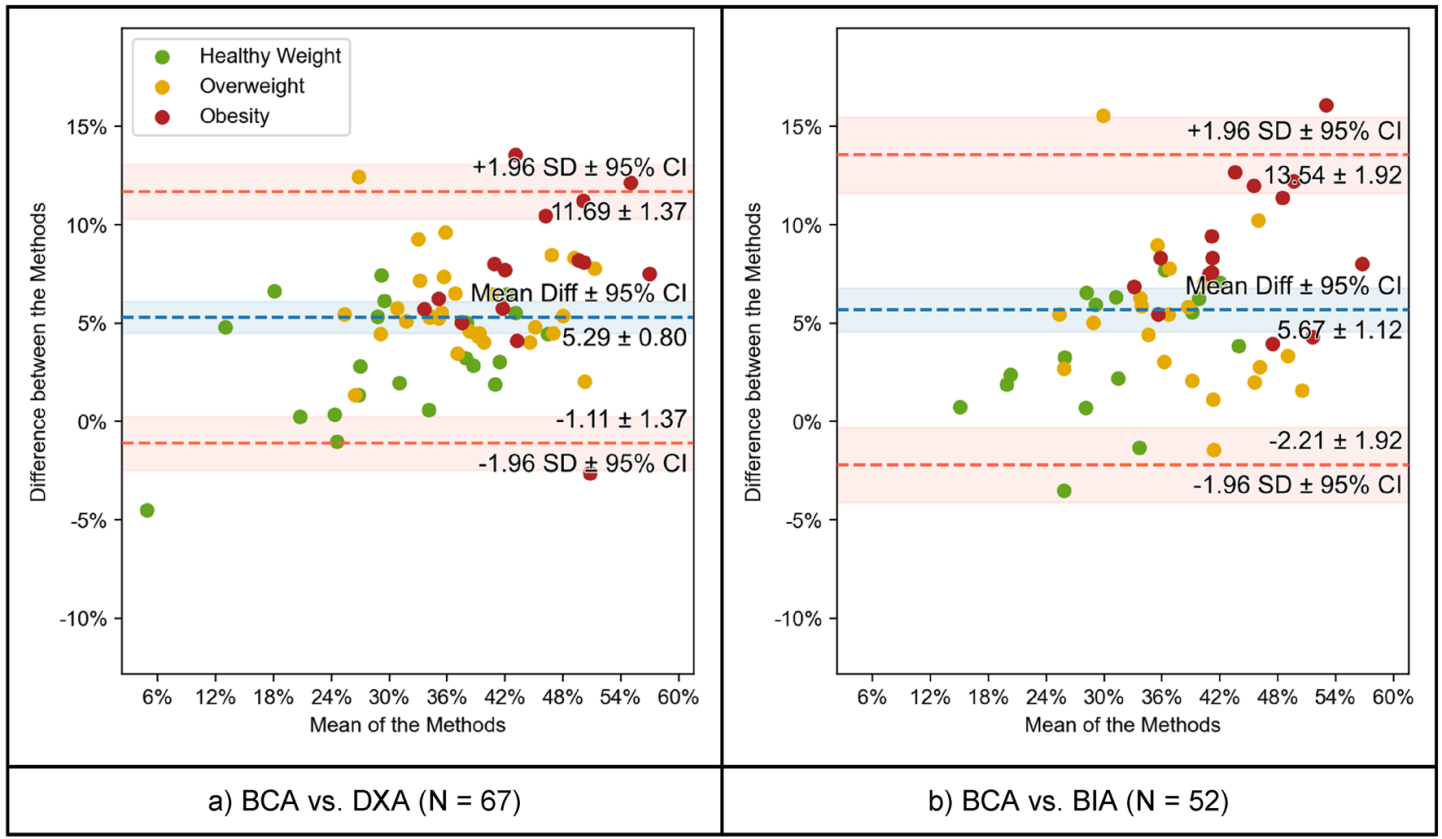
Comparison of BFR between BCA vs. DXA (left) and BCA vs. BIA (right) using Bland–Altman (or mean-difference) plots. Each data point has been colored according to the BMI category of the patient it represents. The mean difference and the limits of agreement are shown in blue and red, respectively, together with their 95% confidence intervals.

**Figure 5:**
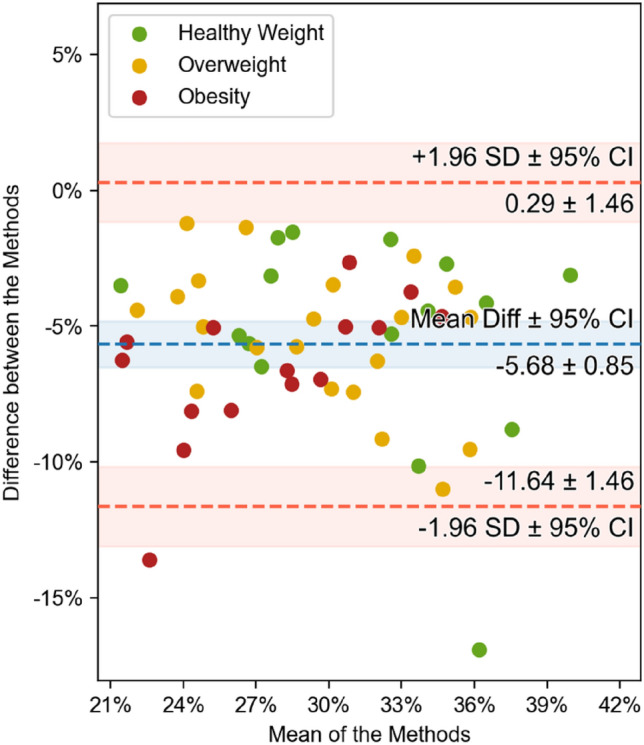
Comparison of SMR between BCA and BIA (N = 52) using Bland–Altman (or mean-difference) plots. Each data point has been colored according to the BMI category of the patient it represents. The mean difference and the limits of agreement are shown in blue and red, respectively, together with their 95% confidence intervals.

The SMR is compared between BCA and BIA and the results showed a clear trend among all BMI-groups, as presented in Fig. 3: r = 0.81, ρC = 0.49. The intermodal comparison between BCA and BIA shows a correlation between the modalities: BMI-group 1: r = 0.75, ρC = 0.49; BMI-group 2: r = 0.86, ρC = 0.49; BMI-group 3: r = 0.88, ρC = 0.39. Figure 5 shows the same relation, although weaker, as displayed in the Bland–Altman plots comparing BFR (Fig. 4). Obese patients tend to differ more from the mean-difference of − 5.68 ± 0.85% towards the lower standard deviation. For healthy weight patients, there is a smaller difference both between the various method results and when comparing BFR. Moreover, the boxplots-analysis of the subgroups show that the distribution of SMR shrinks with an increasing BMI.

## Discussion

The purpose of this study was to quantify the agreement between the results across different BC evaluations obtained by DXA, BIA and CT-based BCA.

### DXA vs BIA

The analysis showed a strong correlation between DXA and BIA with respect to BFR and SMR measured across all BMI-groups.

Previous studies compared BIA and DXA to assess body composition in various populations and found similar results. Beeson et al.^10^ compared BIA with DXA in diabetes patients and found that FM, FM percentage, and FFM were highly correlated (r = 0.96, r = 0.91, and r = 0.95). Furthermore, the Bland–Altman analysis comparing the difference (DXA–BIA) versus average of DXA and BIA results showed a general agreement between the two methods (Supplementary Fig. 1). It was concluded that BIA may provide valid measures of FM, percentage of FM, and FFM, and could be used as a practical tool for the assessment of body composition in diabetics. In addition, Fürstenberg and Davenport et al.^11^ analyzed BIA and DXA for the assessment of whole-body and segmental body composition in hemodialysis patients. Comparing the difference versus the average of DXA and BIA, it was found that the whole-body FM and LM (lean mass) measured by the two methods were highly correlated (r = 0.92 and r = 0.93 in Bland–Altman analysis). The results of previous studies are in agreement with our results, therefore indicating that the methods are interchangeable.

### DXA and BIA vs BCA

As shown in Fig. 2, BCA showed a strong correlation with DXA and BIA regarding BFR. However, either BCA slightly overestimated BFR compared to DXA and BIA or DXA and BIA underestimated BFR. A study by Bredella et al.^12^ stated that the level of hydration can alter the validity of DXA-derived estimates of body composition. The hydration status in tumor patients varies and the percentage of water may sink in overweight or obese patients^12,13^. Compared to healthy weight patients, the overweight cohort and obese cohort showed a weaker correlation between the techniques. This trend continues when comparing BCA with BIA, where the correlation is strong as well but decreased in subjects with increased BMI. Figure 2 visualizes this discrepancy well, as the data points representing BMI-group 1 patients show the smallest difference between the methods compared with BCA (i.e., closer to 0% difference) while patients of BMI-group 2 and, moreover, of group 3 display an increased difference between methods. Therefore, the Bland–Altman plots give an impression how BCA overestimated or how DXA and BIA underestimated BFR, respectively, and that this discrepancy grows with increased BMI.

The SMR compared between BCA and BIA showed a clear trend among all BMI-groups. Figure 3 shows that BIA tended to overestimate SMR compared to BCA or that BCA underestimated SMR compared to BIA. As described in “Crossing perspectives: the importance of time-efficiency for patients” section, the Bland–Altman plot outlines the disparities between the three BMI-groups and how these disparities grow with increasing BMI: although all data points range around a mean difference of − 5.68 ± 0.85%, the SMR-measurement for obese patients differs substantially more than it is the case for healthy weight-patients (Fig. 5).

According to the literature, moderate exercise before BIA measurements leads to an overestimation of FFM and an underestimation of body fat proportion due to reduced impedance^14^. It is advised not to perform BIA for several hours after moderately or highly intense exercise since a 90–120 min moderate intensity exercise before BIA measurement can lead to a significant overestimation of FFM of more than 10 kg, leading to a significant underestimation of body fat^15,16^.

Furthermore, in a Brazilian study, Pimentel et al.^17^ observed that in overweight women, BIA overestimated the percentage body fat but underestimated it in obese women. The overestimation of body fat in the overweight cohort and underestimation in the healthy weight and obese cohorts in BIA correlates with our findings. Interestingly, the correlation was the highest in the obese and healthy weight cohorts and the weakest in the overweight cohort.

Another aspect is that BCA exclusively captures the body regions scanned by CT. Extremities positioned outside the scanned area are not measured by BCA, and thus explain the lower SMR. On the other hand, the BFR increases because a large portion of the scan is made up of the pendulous abdomen, especially if the patient is overweight or obese. Since the BFR is computed as the percentage of fat voxels, and a smaller volume is scanned compared to DXA and BIA (caused by the CT protocol), the abdominal fat becomes of greater relevance. To further investigate this issue, we ran a test with BCA performed only on abdominal CT scans. Supplementary Fig. 2 shows that this effect becomes even more perspicuous when only abdominal CT-scans are used and supports this hypothesis. Figure 1 shows that below the thigh region, a smaller amount of fat can be assumed. This assumption is supported by Fig. 2b,c, where the fat overestimation is even stronger in overweight and obese patients. Physicians must therefore know about this technical factor in the BCA-method. However, this fact does not curtail the method compared to BIA or DXA. It can be further explained by the ultimately different measuring methods: BCA, with CT-imaging as the underlying method, currently only captures the torso with truncated parts of the neck and the extremities. DXA is an actual whole-body imaging method and correlates best with BIA, which also uses all parts of the body. Still, BIA is a non-imaging method which relies on the alterations of an electric current, with several potentially interfering factors. These factors can especially be prevalent in seriously ill patients. The ensuing overcoverage of the relative body fat and undercoverage of muscle tissue in relation to that explains the lower levels of correlation in Fig. 2d for example.

Surely, deep learning-based (DL) BCA performed on CT-scans is a novel method in clinical and experimental medicine and reference curves should be used to check the derived measurements for plausibility or comparability^18^.

To approach that problem, single parameters like VAT could be compared between the two methods (BCA and DXA). These parameters were not releasable in the case of DXA-reports. Ultimately, CT-scans with full body protocols have to be employed for comparison. To test this, another study cohort has to be used since full body CT-scanning is not indicated for these patients.

Because sex has a considerable effect on BC, we itemized the cohort into sex-related groups which clearly illustrated the different distribution of BFR between men and women and confirmed the observed correlations between the methods regarding sex (e.g. “Body fat ratio” section and Supplementary Fig. 3).

### Challenges in comparing BCA

When comparing different technologies, accuracy can be rather difficult to compare for several reasons. First, there is no ground truth available. Using physical phantoms is one way to assess accuracy, but anatomical variations lead to different measurement errors. Second, not all methods measure the same thing, so even if two technologies show a strong correlation, there may be a risk of bias if they measure different physical objects. For example, BCA measures body fat volume, while DXA measures body fat mass. Using a constant density factor to convert the measurements may not always be accurate. For this reason, this study compares the ratios of body fat and skeletal muscle in order to avoid conversion factors and further calculations to compare different units.

As previously discussed, the analysis of different regions of the body yields over- and underestimations, making it hard to find a conversion factor between the methods. One way to obviate this problem is to compute BCA on head-to-toe whole body-CT-scans, and to estimate what amount of fat and muscle these accessorily scanned body regions would add to the total. Also, with the results generated from this study, a correction factor can be implemented to compare a patient's BC results between the three methods in clinical practice rather than converting results between the three methods. This corrective factor must take into account the patient’s BMI-group, as results diverge more between methods with increasing BMI (e.g., “Body fat ratio”, “Skeletal muscle ratio” sections).

DXA assumes that the hydration of fat-free tissue remains constant at 73%^19^. However, hydration status varies from 67 to 85%^20^. If a subject contains more than the average amount of water, e.g., due to ascites or oedema, some DXA scanners will overestimate the fat content. Although a ± 5% range of fat-free tissue hydration does not significantly alter the total percentage of fat, severe overhydration, may affect the resulting percentage of fat, which is especially relevant in cancer patients.

We compared the three analyzed methods (Table 2) underlining that, once a high correlation is given, the methods are interchangeable. However, the methods have their strengths and weaknesses in different areas. For our neuroendocrine tumor cohort, CT- based BCA bears several advantages compared to DXA and BIA since it is a “one-stop-shop solution” as part of the staging process. Furthermore, BCA shows high tissue differentiation and offers fast results independent of the patient’s condition. The BCA network is also trained and capable of computing contrast-enhanced as well as non-contrast enhanced CT-scans (e.g., “Deep learning architecture” section), which further reduces contraindications, e.g., for patients with contrast agent allergy.

**Table 2:** Arguments for and against the methods for BC assessment are summarized and compared to DL-based BCA performed on routine staging CT-scans.

	BCA	DXA	BIA
Diagnostic effort	**As part of staging**	**Additional examination**	**Additional examination**
Availability	High	Moderate	Moderate
Cost savings	Costs saved	Additional costs	Additional costs
Radiation exposure	None [10–15 mSv]	4–5 µSv	None
Time consumption	1.4 min [5–7 min]	5–15 min	1 min
Cooperation required by patients	None	None	Safe foothold
(Adipose) tissue differentiation	High: anatomic level differentiation	Low: limited differentiation to extremities and torso	None

The row highlighted in bold points out the intended integration of the method into the diagnostic process relating to the rows following underneath. The details written in square brackets state the respective information concerning the CT-scan, which is used for BCA.

### Crossing perspectives: the importance of time-efficiency for patients

Physicians often tend to compare methods solely based on hard facts, such as radiation dosage, costs, or time consumption. These are indeed important factors; however, these factors are not sufficient when it comes to direct patient care and the effects on the individual treated. Quality of Life (QoL) is considered an important clinical endpoint, especially for long-lasting diseases such as cancer^21^ and must be considered in this regard. For example, the time spent hospitalized can be seen as a further reduction of the time the patient is able to spend with their family and friends, which is a major factor for improvement of cancer patients’ QoL^22,23^. Even with short examination times of the diagnostic method, the time until the patient arrives at the appropriate hospital facility and is examined can be substantially longer for the patient. Cutting out one to two whole exams, and nevertheless gathering the same information (together with further imaging information) via CT-based BCA, reduces the waiting time spent in hospital hallways and could improve the patient’s QoL.

### Limitations

Although our study demonstrates that CT-BCA can be used to efficiently assess the patient’s nutrition status, it is not free of limitations. In a well-regulated prospective setting, these factors could have been excluded and the efficiency could have been improved. However, we wanted our system to be tested in a realistic clinical setting and to provide reliable results even with slight variations that occur in daily clinical practice. Moreover, because of the CT protocol’s curtailment from the base of the skull to the mid thighs, SAT and muscle tissue was measured in a standardized manner but could not be measured in total. A whole body-scan “from head to toe” could solve this data deficit but lacks indication in clinical practice. Finally, we could only demonstrate the overall efficacy in a circumscribed study population at a single center; multi-center follow-up studies with different diseases are needed to validate whether our approach can be generalized to other entities.

### Future outlook

Body composition analysis plays an important role especially in slowly proliferating cancer since the change in muscle and fat tissue during the course of the disease could be indicative of cancer progression or stability^24^. A higher BMI is associated with a higher risk of cancer. Paradoxically, Overweight and grade I obese patients often have a paradoxically lower risk of overall mortality after a cancer diagnosis, a phenomenon this is called the “obesity paradox” or rather “BMI paradox”^25^. Since BMI neither distinguishes muscle from adipose tissue nor describes adipose tissue distribution, the sensitivity in diagnosing obesity is weak leading to high misclassification rates. Therefore, body composition analysis should be used to detect an early increase in adipose tissue and reduction in muscle which can lead to a state of sarcopenic obesity^26,27^.

Other imaging methods might also be a valuable extension and improvement to BC measurement, such as magnetic resonance imaging (MRI) which would eradicate radiation uptake as a whole and offer even more imaging information, especially of soft tissues. Despite these advantages, MRI is a less available technique in most nations, especially in less developed countries^7^. Yet, developing DL-algorithms apt for MRI necessitates the integration of much more imaging data points.

Currently, CT and MRI are still considered the gold standard for research and diagnostic purposes with DXA as the preferred alternative for research and clinical use. BIA on the other hand is considered a portable alternative to DXA^28^. With an automated tool like the applied BCA at hand, the gold standard for BC measurement becomes increasingly applicable in a wide set of ambits.

The DL-based approach^29^ allows the BCA-network to process mostly unstructured CT-data and continuously improve its calculations when it is being trained with an increasing amount of input-data compared to non-DL-based solutions.

Furthermore, the implementation of BCA in cancer patients and the evaluation of change in different tissues could be useful for the detection of cancer progression and disease control rate. This measurement-technique, i.e., BCA, is especially useful in NET-patients due to these tumors indolent course compared to entities such as pancreatic adenocarcinoma or hepatic cancer.

The emergence of automated techniques to quantify body composition will allow for rapid and early intervention, especially of high-risk patients. The integration of body composition measurement into oncology offers a tremendous promise to help patients with cancer live longer and healthier lives and experience enhanced QoL. These factors should be focused on by future multicenter studies with larger cohorts and correlation to clinical endpoints.

## Materials and methods

### Study design and study population

Patients were identified from our prospective NET database at the European Neuroendocrine Tumor Society (ENETS) Center of Excellence, Department of Endocrinology, Diabetes and Metabolism at the University Hospital Essen. Eligible patients included those with histologically confirmed, well-differentiated NET who were treated at our Department between February 2019 and October 2021, with all records located at our endocrine tumor center. All patients underwent contrast-enhanced [68Ga]-DOTATOC-PET/CT at initial presentation and subsequent follow-up visits. Patients with incomplete data and with PET/CT-scans more than 15 days before or after DXA and BIA exams were excluded from further analysis. To ensure consistency, scheduling of visits as well as indication for therapies was determined according to ESMO (European Society for Medical Oncology) guidelines^8^ by an experienced, multidisciplinary tumor board. All staging scans were performed in-house at our center. Whole-body PET/CT-scans were obtained as part of the staging process, and DL-based BCA was applied retrospectively. Additionally, DXA- and BIA-scans were obtained within 1–3 days during the same hospital stay before or after CT-imaging from which a BC-report was calculated. Patient characteristics are listed in detail in Table 1. For this study, patients who underwent a staging at our clinic via PET/CT received DXA and BIA at the same appointment for intermodal comparison. Patient data was analyzed retrospectively.

### Ethics statement

Written informed patient consent and approval for anonymized data collection and analysis was obtained upon admission to our institution. The study was approved by the ethics committee of the University Hospital Essen (ID: 18-8367-BO).

### [68Ga]-DOTATOC PET/CT-scan

[68Ga]-DOTATOC PET/CT was performed as a whole body-protocol with a Biograph mCT 128 (Siemens Healthcare GmbH, Erlangen, Germany) 41 ± 18 min after intravenous injection of [68Ga]-DOTATOC radionuclide agents with a mean activity of 65 ± 11 MBq (Megabecquerel). The CT scan was acquired with an automatic dose modulation at 120 kV, 210 mAs_eff_, collimation 128 × 0.6 mm, pitch 0.8, slice thickness of 5.0 mm, and 5.0 mm increment, ranging from the skull base to the mid-thigh. Intravenous administration of an iodinated contrast agent (Xenetix 300; Guerbet GmbH, Sulzbach, Germany) was used and a whole body-scan obtained in the portal venous phase with a contrast medium administration delay of 70 s. Patients were examined in the supine position with elevated arms. Phase attenuation correction was based on the portal venous phase whole body-CT-scan. PET acquisition was performed in five to seven bed positions with an acquisition time of 2 min per bed position (FOV 21.8 cm, matrix size 256 × 256). An iterative ordered subsets expectation maximization (OSEM) algorithm with three iterations, 21 subsets and a Gaussian filter of 4 mm was used for reconstruction. PET attenuation correction was based on the portal venous phase of the whole-body CT-scan.

Only the CT-imaging data component with 5 mm reconstruction using the soft tissue kernel was utilized by the DL-network. The average PET/CT examination time was 18 ± 2 min.

### DXA

The Lunar Prodigy (GE Healthcare, USA), a narrow fan-beam densitometer, was used for BC measurement via DXA. The Lunar Body Composition Software option applied on the Lunar DXA bone densitometer measured the regional and whole-body bone mineral density (BMD), lean and fat tissue mass (FM). Additionally, it calculated derivative values of bone mineral content (BMC), soft tissue mass, regional soft tissue mass, total soft tissue mass, FFM (fat-free mass), regional/total soft tissue mass ratio, percentual fat mass, regional percentual fat mass, total body percentage fat mass, Android percentual fat mass, Gynoid percentual fat mass, Android/Gynoid fat mass ratio (A/G ratio) and BMI. Total fat mass was considered as the relevant output of this method and used for intermodal comparison.

### BIA

The medical BCA analyzer, seca-mBCA 515 (seca GmbH, Germany) used multi-frequency 8-point stand-on bioelectrical impedance analysis to measure total body water by applying an electrical current of 100 µA to the body. The drop in voltage between sensor electrodes at the hands and feet was used to determine total body water. The software calculates fat mass, fat free mass, skeletal muscle mass and VAT (visceral adipose tissue) volume from total body water, weight, height, age and gender. The BIA device measured at 20 frequencies, ranging from 1 to 1000 kHz. Patients were scanned once in the standing position, with four electrodes at the feet and four electrodes at the hands. The scan lasted 60 s. Total fat mass and skeletal muscle mass were considered as the relevant output of this method and used for intermodal comparison.

### Deep learning architecture

The CT-based BCA was provided by the in-house body composition analysis DL-network, which is an evolution from the system described in Koitka et al.^29^. The DL system utilized a multi-resolution U-Net 3D network to segment the body into semantic regions. Technical details of the respective methodology are disclosed in Koitka et al.^29^. In this study, the previously developed body composition analysis system was employed, which was trained on 300 CT imaging studies in total (100 abdominal, 100 thoracic, 50 head/neck, 50 whole body-scans) and tested on a separate test-dataset composed of 20 thoracic, 20 abdominal, 10 head/neck and 10 whole body-CT-scans all annotated manually by eight experienced human readers. This dataset was divided into two equal sets, one consisting of non-contrast-enhanced scans and the other consisting of contrast-enhanced CT-scans that either excluded the head and neck or captured the whole body, according to the hospital's common CT requests. The scans in both sets were randomly selected without any specific inclusion or exclusion criteria.

### Automated tissue-quantification

Body composition was calculated automatically by the pre-trained DL-network on all 74 CT-scans. Seven volumes for different body compartments were examined. This approach allowed the volume of five different adipose tissue biomarkers to be quantified: Subcutaneous Adipose Tissue (SAT), VAT, Intermuscular Adipose Tissue (IMAT), Epicardial Adipose Tissue (EAT) and Paracardial Adipose Tissue (PAT). Additionally, muscular tissue and bone volumes were also computed. The applied DL-network calculates specific tissues by thresholding the HU to a specific intensity range in a given semantic body region. Adipose and muscular tissues were identified using known Hounsfield unit thresholds, namely − 190 to − 30 HU for adipose tissue and − 29 to 150 HU for muscular tissue^30^, and then subclassified using the semantic body regions predicted by the DL system. Due to the scanning curtailment given by the CT protocol, IMAT and SAT could not be scanned to the full extent (see “[68Ga]-DOTATOC PET/CT-scan” section). Segmented regions like the pericardium can also be used directly to measure the total volume enclosed by the pericardial sac. Further detail about the anatomical differentiation in the segmentation process is given in Kroll et al.^31^ and Koitka et al.^29^. For this use case, BCA had two relevant outputs: a complete report showing the volumes of the above-mentioned biomarkers, and a tissue segmentation presenting their positions. Since DXA and BIA compute body fat percentages according to the mass, and there is not a clear conversion between volume and mass for fat and muscle tissue, these volumes were discarded, and the tissue segmentation was used instead. The Body Fat Ratio (BFR) was computed by considering the amount of voxels identified as adipose tissue (i.e., the union of the voxels belonging to the SAT, VAT, IMAT, EAT and PAT classes) divided by the total amount of voxels present in the CT scan and stated as a percentage. The total amount of voxels was defined as the patient’s body's voxels specifically excluding abdominal air (HU Threshold: − 1024 to − 800), to reduce inconsistencies. Similarly, the Skeletal Muscle Ratio (SMR) was computed by considering the amount of voxels identified as muscular tissue divided by the total amount of voxels and stated in percent as well.

### Statistical analysis

Statistical analysis was conducted using Python 3.8 and the SciPy packages (version 1.6.1)^32^. Pearson rank correlations (*r*) were calculated to determine the relationships between the three compared methods (BCA, DXA and BIA) regarding the relevant tissue types. The p-value for testing non-correlation for Pearson’s r was also computed, which relies on the assumption that each dataset is normally distributed, and a Shapiro–Wilk test was conducted to confirm a Gaussian distribution.

To further evaluate the reproducibility of this new method, and the agreement between BCA, DXA and BIA respectively, the concordance correlation coefficient of Lin (*ρC*) was used^33^, as well as Bland–Altman-plotting^34^ for visual statistical comparison.

### BMI grouping

The patients were analyzed with respect to their age, gender, and BMI-group to establish comparability. BMI-grouping divided the patients into the three following groups based on the collectives’ given distribution: BMI-group 1, healthy weight: 18.5 to < 25.0 kg/m^2^, n = 22; BMI-group 2, overweight: 25.0 to < 30 kg/m^2^, n = 35; BMI-group 3, obese: ≥ 30 kg/m^2^, n = 17. Interestingly, none of the patients were underweight, i.e., BMI < 18.5 kg/m^2^.

## Conclusion

CT-based BCA obtained from regular staging exams produces precise and stable results in patients suffering from GEP-NET comparable to those obtained via DXA and BIA. BCA implemented as a substitute for these modalities could substantially improve the quality of life for the individual patient alongside the reduction of costs, radiation dose and consumption of resources in general. Clinical correlation to changes in BCA and tumor reduction or progression is needed in multicenter studies.

## Supplementary Information

Supplementary Figure 1.

Supplementary Figure 2.

Supplementary Figure 3.

## Data Availability

The data analyzed in this study is not publicly available due to privacy and security concerns. The data may be shared with a third party upon execution of data sharing agreement for reasonable requests, such requests should be addressed to L.K. (e-mail: Lennard.kroll@uk-essen.de) or A.M.

## Code availability

The code may be made available from the authors upon reasonable request subject to permission and approval from the corresponding organizations and institutional review boards.
