# Supplementary material for: CT-derived body composition analysis could possibly replace DXA and BIA to monitor NET-patients
Source: Sci Rep. 2022 Aug 4;12:13419. doi: 10.1038/s41598-022-17611-3 (PMC9352897; doi:10.1038/s41598-022-17611-3)
Supplement: Supplementary file 1 — Supplementary Figure 1. [file 41598_2022_17611_MOESM1_ESM.pdf]

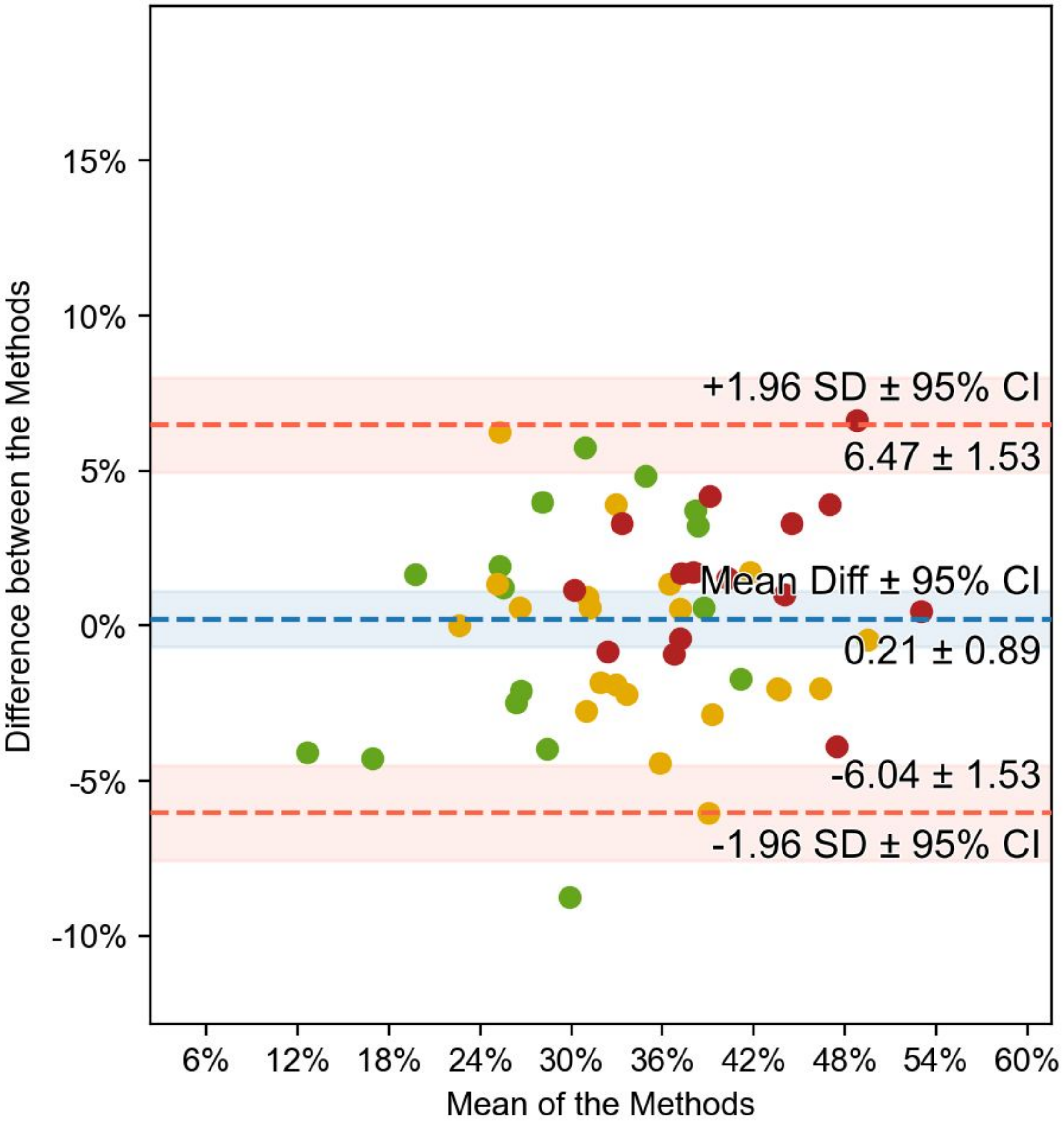

Supplementary Figure 1: Comparison of BFR between DXA vs. BIA using Bland-Altman (or mean-difference) plots. Each data point has been colored according to the BMI category of the patient it represents. The mean difference and the limits of agreement are shown in blue and red, respectively, together with their 95% confidence intervals.
