# Supplementary material for: CT-derived body composition analysis could possibly replace DXA and BIA to monitor NET-patients
Source: Sci Rep. 2022 Aug 4;12:13419. doi: 10.1038/s41598-022-17611-3 (PMC9352897; doi:10.1038/s41598-022-17611-3)
Supplement: Supplementary file 2 — Supplementary Figure 2. [file 41598_2022_17611_MOESM2_ESM.pdf]

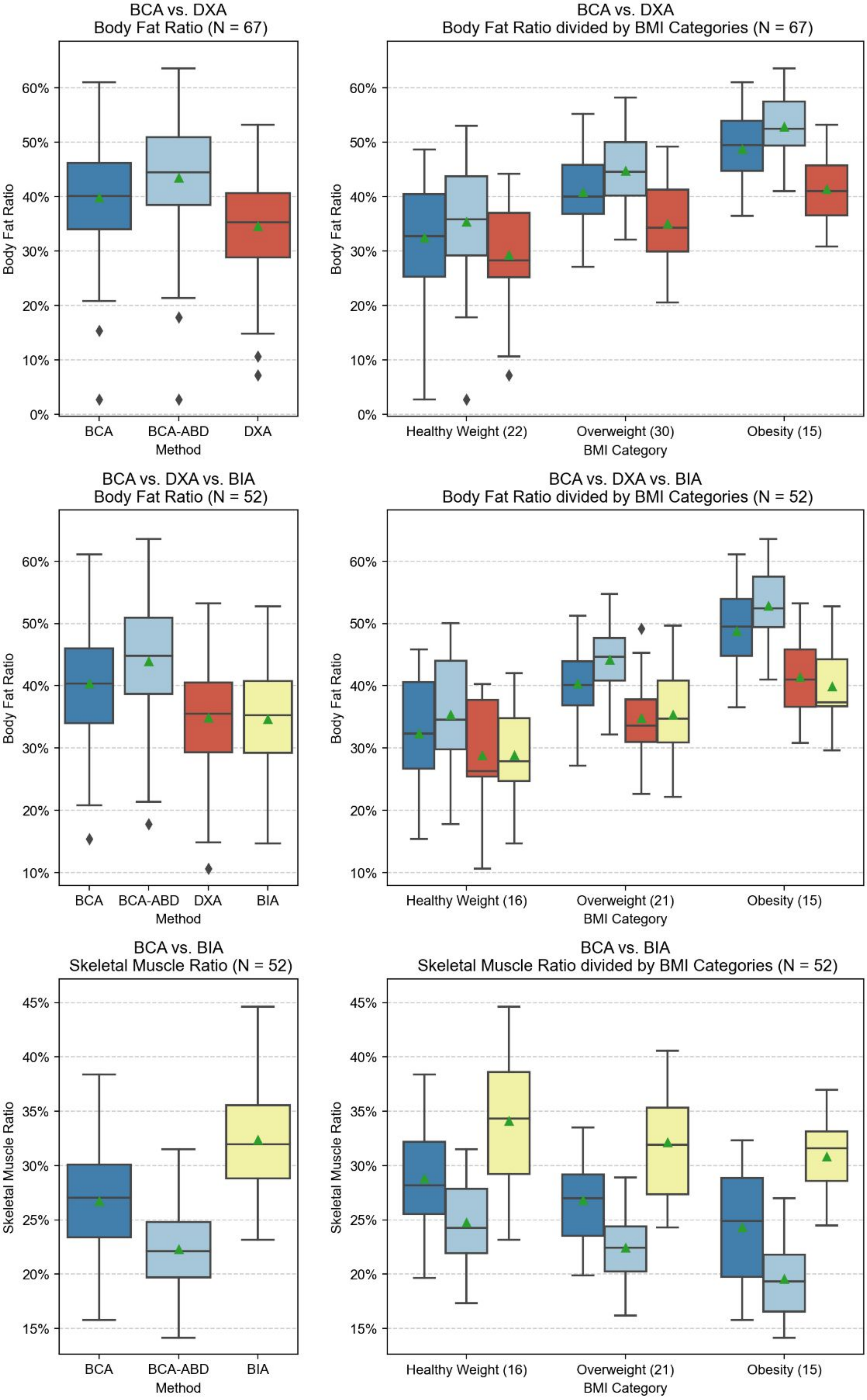

Supplementary Figure 2: Comparison of BFR and SMR between BCA performed on whole body-CT (blue), BCA performed on abdominal CT (light blue), DXA (red) and BIA (yellow). The boxplots represent the distribution of the patient's measurements, the mean is indicated with a green triangle and the outliers are indicated with a rhombus.
