# Supplementary material for: CT-derived body composition analysis could possibly replace DXA and BIA to monitor NET-patients
Source: Sci Rep. 2022 Aug 4;12:13419. doi: 10.1038/s41598-022-17611-3 (PMC9352897; doi:10.1038/s41598-022-17611-3)
Supplement: Supplementary file 3 — Supplementary Figure 3. [file 41598_2022_17611_MOESM3_ESM.pdf]

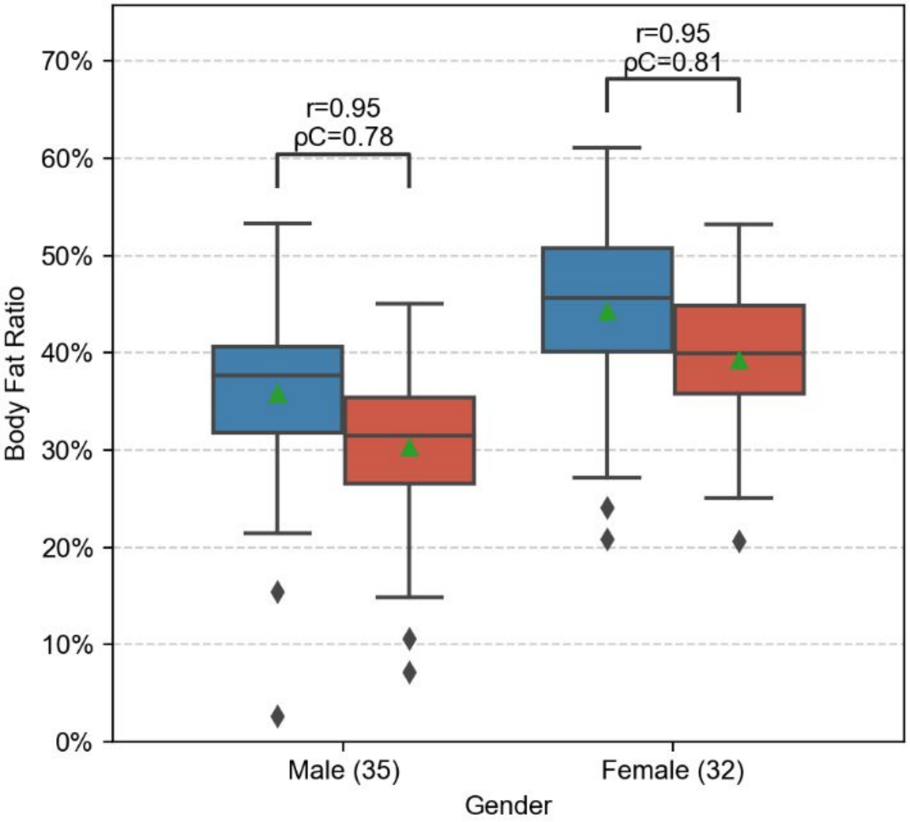

a) BCA vs. DXA, comparison of body fat ratio (N = 67) between male and female patients

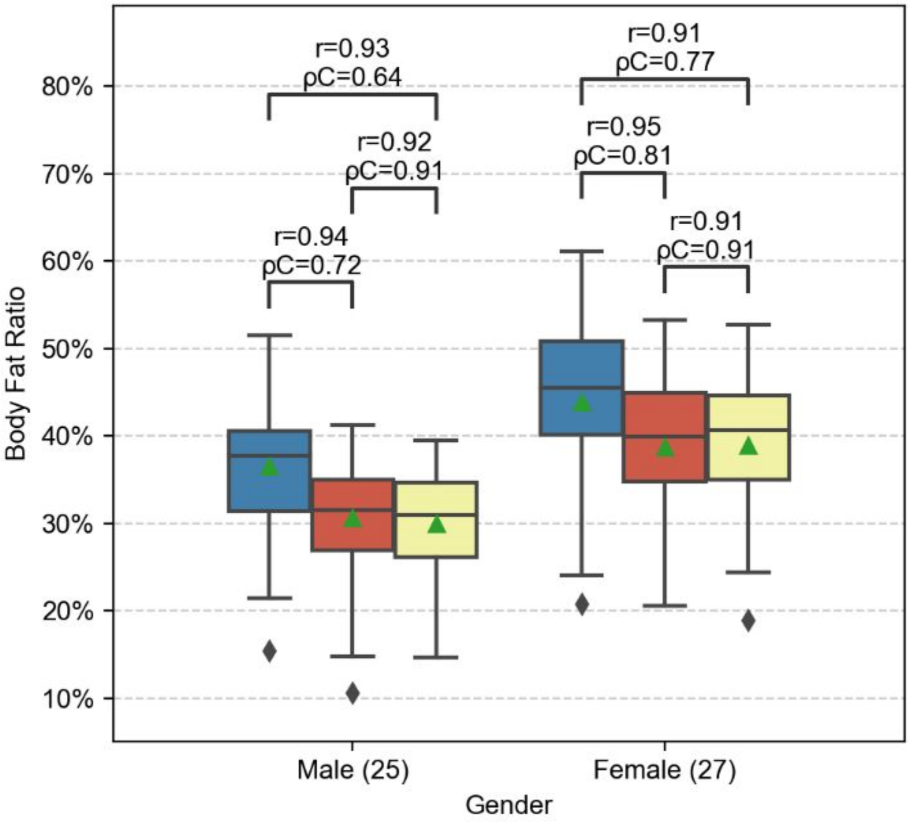

b) BCA vs. DXA vs. BIA, comparison of body fat ratio (N = 52) between male and female patients

Supplementary Figure 3: Comparison of BFR between BCA (blue), DXA (red) and BIA (yellow) itemized into sex-related groups. In plot a, BCA and DXA are compared separately because more patients received PET/CT- and DXA scans. Patients with all three measurements available are compared in plot b. The boxplots represent the distribution of the patient's BC measurements. The mean is indicated with a green triangle and the outliers are indicated with a rhombus. The samples are compared using Pearson's r correlation coefficient (r) and Lin's concordance correlation coefficient (ρC).
